# Effects of Air Injection on the Dynamic Characteristics of Sediment Deposition and Erosion Distribution in Pump-Turbine Runners

**DOI:** 10.3390/s25196192

**Published:** 2025-10-06

**Authors:** Haowen Liu, Xin Yang, Yanjun He, Jiaxing Lu, Liang Jiang

**Affiliations:** 1 Key Laboratory of Fluid and Power Machinery, Ministry of Education, Xihua University, Chengdu 610039, China; haowenliu@xhu.edu.cn (H.L.); yx999511@163.com (X.Y.); yanjunhe1203@163.com (Y.H.); jiangjohn0403@163.com (L.J.); 2 School of Energy and Power Engineering, Xihua University, Chengdu 610039, China; 3 Key Laboratory of Fluid Machinery and Engineering, Xihua University, Chengdu 610039, China

**Keywords:** pump-turbine, air injection, sediment erosion, numerical simulation

## Abstract

As the core equipment of pumped storage power stations, the erosion and wear of pump-turbine runner blades have become critical issues affecting the operational efficiency and service life of units. To mitigate particle impact energy and surface wear of the runner blades, this study proposes the installation of aeration short pipes with a diameter of 4 mm and a length of 20 mm at the adjustable guide vanes and the main shaft, aiming to investigate the influence of air injection on erosion characteristics. Numerical simulations demonstrate that air injection significantly alters the flow characteristics in the runner region and establishes a coupling relationship between erosion distribution and different aeration concentrations and injection methods. Guide vane aeration effectively reduces sediment-induced erosion on the runner crown and blade working surface, while simultaneously leading to more localized erosion on the crown and band. In contrast, main shaft aeration increases erosion on the crown and blade working surface, and under high aeration concentrations, the erosion region exhibits a significant expansion trend.

## 1. Introduction

The pump-turbine is the core reversible unit of pumped storage power stations, integrating the dual functions of both a pump and a turbine. In pumping mode, it lifts water from the lower reservoir to the upper reservoir for energy storage; in generating mode, it efficiently converts the potential energy of the upper reservoir into electricity. With its capabilities of peak shaving, valley filling, frequency regulation, and phase regulation, the pump-turbine plays a vital role in ensuring the safe and stable operation of power systems [1]. To accommodate the rapid large-scale development of renewable energy and to safeguard the security and stability of power systems, pumped storage power stations are expected to play an increasingly significant role [2,3].

Pump-turbines often operate under complex conditions where sediment particles cause severe wear on guide vanes and runners [4]. Large fluctuations in operating head also force the units to run under off-design conditions, making high-velocity flows prone to cavitation [5]. Cavitation not only damages metal surfaces directly but also accelerates sediment erosion, leading to scratches that aggravate flow instabilities and further worsen cavitation [6]. Surveys indicate that about 40% of hydropower facilities in China face serious cavitation and sediment erosion issues, resulting in efficiency loss, increased vibration and noise, higher maintenance costs, and risks to safe and economical operation [7].

Extensive research has been conducted by scholars worldwide through experiments and numerical simulations to investigate the influencing factors of sediment erosion, including flow velocity, impact angle, particle size, sediment concentration, and material properties. However, effective engineering measures to fundamentally mitigate erosion remain limited.

Sharma et al. [8] investigated Francis turbines in the Himalayan region under varying conditions and sediment concentrations, and found that wear rate increased almost linearly with sediment concentration, with runner erosion exceeding that of fixed and movable guide vanes by over 90% and 79%, respectively. Wang Y.P. et al. [9] employed the Mixture multiphase flow model, RNG k−ε turbulence model, and SIMPLEC algorithm to simulate two-phase flow in a centrifugal pump, analyzing the effects of particle size and concentration on pressure distribution, solid-phase distribution, and overall performance. Wang P. et al. [10] designed rotating discs with three blades for experimental testing, compared the results with numerical simulations, and confirmed their flow and wear behaviors. Peng G.J. et al. [11] used an Euler–Euler multiphase flow model to simulate slurry flow characteristics at low flow rates with different particle concentrations, showing significant unsteady features. Wang L.Y. [12] adopted the Particle model and the inhomogeneous model in CFX to investigate turbine wear and performance in solid–liquid flows. Sun J. et al. [13] employed the Euler model and RNG k−ε turbulence model to perform unsteady simulations of a bulb turbine unit, analyzing the impact of sediment particles on pressure pulsation. Liu X.B. [14] applied an Euler–Lagrangian turbulence model to simulate sediment erosion in a turbine under low sediment concentrations, accurately predicting the erosion rate and main erosion regions of the guide vanes with good agreement to experimental data. 

Research by Padhy and Saini [15] and Thapa [16] on sediment erosion in Francis turbines showed that the relative velocity between the runner and fluid is a critical factor causing abrasive damage. Peng G.J. et al. [17] applied an Euler–Lagrangian model with the Finnie erosion model to predict sediment erosion of runner blades and guide vanes, and validated the results against field experiments, showing a positive correlation between erosion rate and sediment concentration, with runners experiencing greater erosion than guide vanes. Li L.H. [18] examined the effects of sediment concentration and particle size on Pelton turbine erosion, finding that concentration mainly determined erosion magnitude, while particle size influenced erosion location. Zhang L. [19,20] employed the DPM and standard turbulence model to simulate small-opening operating conditions, revealing the flow and erosion characteristics near guide vanes. Li Y.H. et al. [21] used the Oka model and field data to predict runner erosion in a high-head Francis turbine, showing that off-design small-opening conditions caused vortices and sediment accumulation, increasing runner erosion rates. Cruzatty [22] combined liquid–solid two-phase flow simulations with erosion models to evaluate erosion rates under various operating conditions, finding that erosion sharply increased near full-load conditions, particularly when guide vane openings exceeded 90%. Kang et al. [23] used the DPM to predict runner erosion in Francis turbines under different operating conditions and sediment concentrations, showing that erosion mainly occurred on the pressure side of the blades. Wei X.Y. [24] investigated erosion from a flow field perspective, demonstrating a strong correlation between erosion locations and vortex distribution between blades.

Pan J.L. [25] studied both pump and turbine operating modes of pump-turbines, finding that the impeller was the most vulnerable component, closely related to local high flow velocities. Lu J.X. [26] investigated sediment erosion characteristics of pump-turbine runners under different solid-phase conditions, showing that particle size and concentration significantly affect erosion distribution and intensity, playing a decisive role in wear patterns. Yang Z.Y. [27] combined experiments and numerical simulations to analyze the promoting effect of sediment on cavitation, revealing that particles not only altered the development of local cavitation regions but also intensified their impact on runner walls, thereby increasing wear risk.

Nevertheless, relatively few studies have examined the effects of aeration on sediment erosion in pump-turbines. To better understand the complex three-phase interactions among sediment particles, water, and air inside the runner under sediment-laden conditions, this study combines experimental tests with numerical simulations to investigate the influence of air injection on sediment flow in the pump-turbine runner. The impacts of aeration location and concentration on sediment particle movement, erosion distribution, and erosion intensity are analyzed, providing important theoretical support for erosion mitigation through aeration in pump-turbines.

## 2. Numerical Model

### 2.1. Simulation Setup

To mitigate the particle impact energy, aeration short pipes with a diameter of 4 mm and a length of 20 mm were installed at the guide vane and main shaft positions, aiming to modify the near-wall flow characteristics through air injection. Using the FLUENT software 2020, a comparative analysis was conducted under turbine operating conditions of the pump-turbine to investigate the effects of different aeration locations (guide vane and main shaft) and concentrations on sediment erosion of flow-passing components.

A steady-state clear-water condition was first established for numerical simulations. The working fluid was water at 25 °C with a density of 998 kg/m^3^. The computational domain inlet was set at the volute entrance with a mass flow rate boundary condition, and the outlet was defined at the draft tube exit with a pressure outlet boundary condition. The runner surface was modeled as a rotating wall moving at the runner speed, while all other walls were treated as no-slip walls without surface roughness, and the standard wall function was applied near the wall. The SIMPLE algorithm was used for pressure–velocity coupling, and the second-order upwind scheme was employed for spatial discretization. Convergence was achieved when the residuals dropped below 10^−6^.

The steady-state solution was first obtained and then used as the initial condition for unsteady solid–liquid simulations. In the unsteady simulations, sediment particles were injected at the volute inlet using the Discrete Phase Model (DPM). A time step corresponding to a 2° rotation of the runner (3 × 10^−4^ s) was applied, with a total simulation duration equivalent to 20 runner revolutions (1.2 s).

(1) $$IAVF=\;\frac{q}{Q_{inlet}}$$

where q denotes the volumetric flow rate of the gas (m^3^/s), and $Q_{inlet}$ represents the turbine inlet flow rate, which is 0.022 m^3^/s.

For guide vane aeration, the inlet air volume fraction (IAVF) was set to 0%, 2%, 4%, and 6%, while for main shaft aeration, IAVF was set to 0%, 1%, 2%, and 3%. In the main shaft scheme, ventilation channels were installed to directly inject air into the runner region. Comparative analyses were conducted to evaluate the effects of different guide vane aeration concentrations on sediment erosion of critical flow-passage components. The aeration schemes are summarized in Table 1 and Table 2.

### 2.2. Modeling of the Pump-Turbine

**Figure 1:**
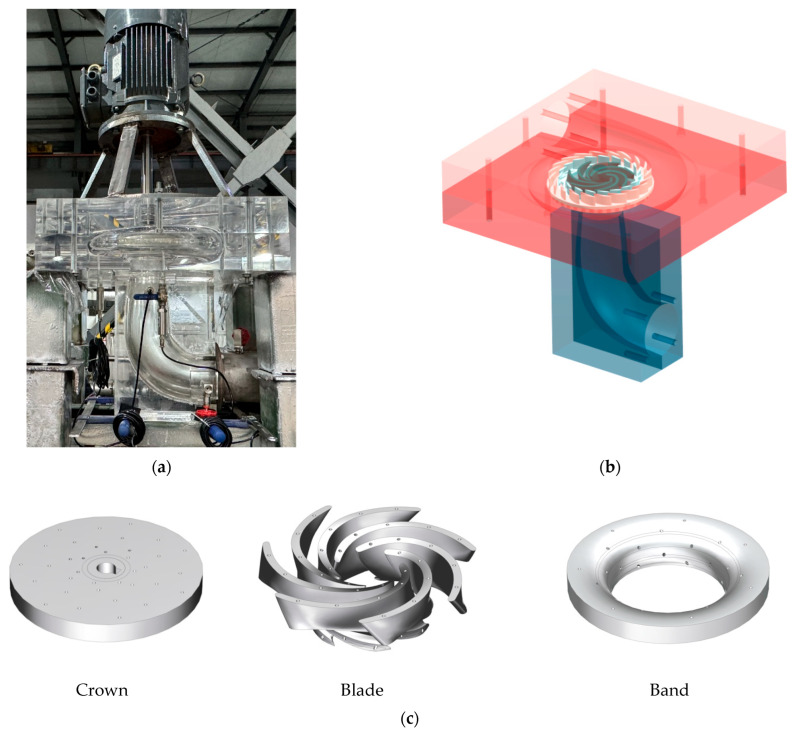
Pump turbine and runner model diagram: (**a**) Photograph of the pump-turbine runner. (**b**) Pump turbine model diagram. (**c**) Runner model diagram.

Based on the model data of the pump-turbine, a three-dimensional model was established, as shown in Figure 1a–c. The main flow-passing components of the turbine consist of five parts: the spiral casing, runner, stay vanes, guide vanes, and draft tube. The model includes 19 guide vanes, 20 stay vanes, and 7 runner blades. Other model parameters are listed in Table 3.

**Figure 2:**
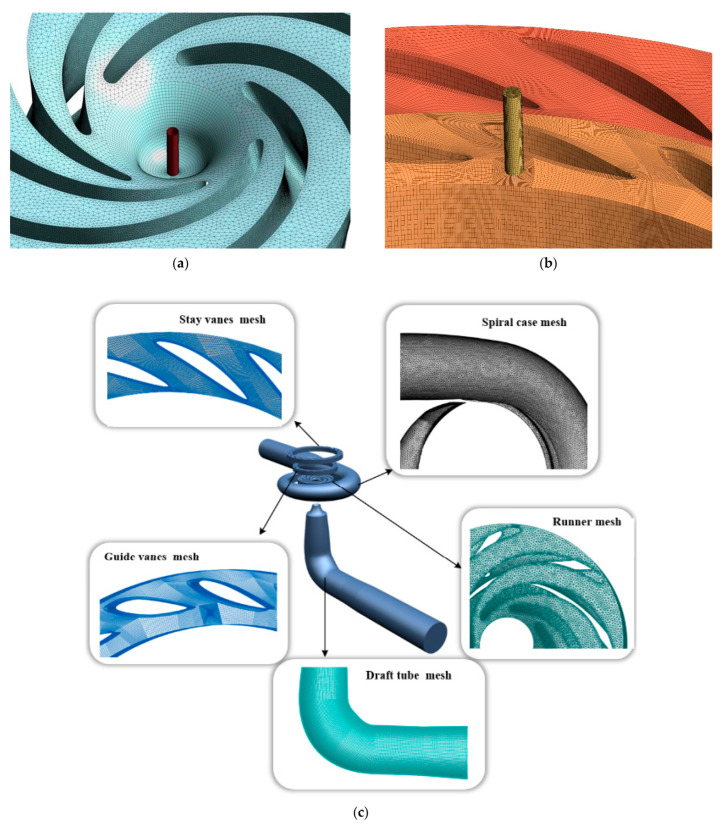
Geometric Model of the Aeration Device for Pump-Turbine and Mesh Model of the Computational Domain. (**a**) Mesh of the aerated guide vanes. (**b**) Mesh of the aerated main shaft. (**c**) Calculate the domain grid model.

For the runner components, an unstructured tetrahedral mesh was generated using CFD ICEM software 2020. The boundary layer was locally refined to improve computational accuracy, and additional mesh refinement was applied to critical wall surfaces, including the guide vane surfaces, runner blades, and draft tube walls. The remaining computational domain was discretized with high-quality structured hexahedral grids. The model and mesh are shown in Figure 2a–c.

**Figure 3:**
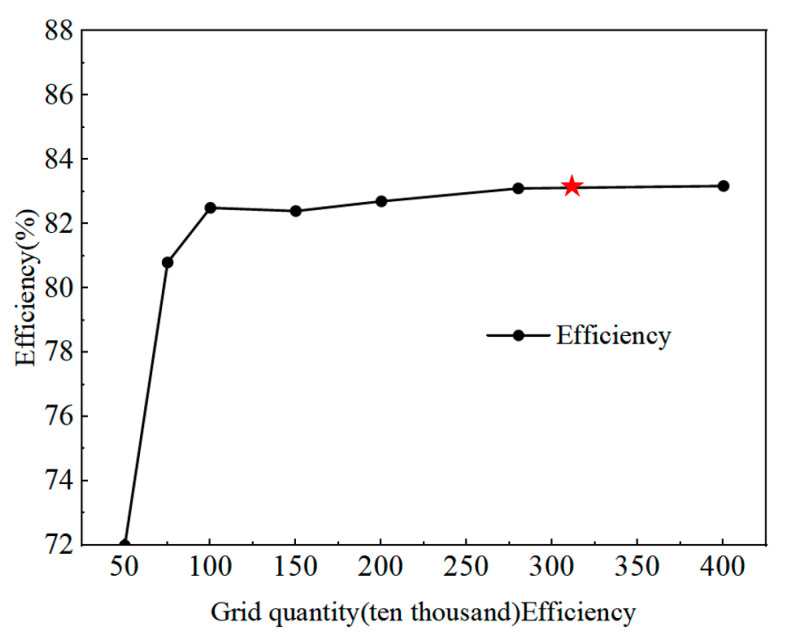
Grid relation verification of hydraulic efficiency of hydraulic turbine.

To reduce the influence of grid density on the computational results, six sets of meshes were generated to perform a grid independence test, as shown in Figure 3. It can be observed that the turbine efficiency increases with the number of grids. When the grid number reaches between 3.0 and 4.0 million, the turbine efficiency no longer shows significant variation, remaining between 82.7% and 82.8%. To ensure computational accuracy while saving resources, a mesh with 3.01 million elements was finally adopted.

### 2.3. Validation of External Characteristic Tests

**Figure 4:**
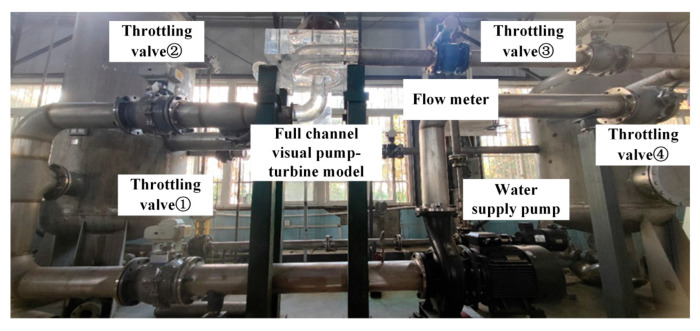
Visualized Pump–Turbine Model Test Bench System (Physical Setup).

**Figure 5:**
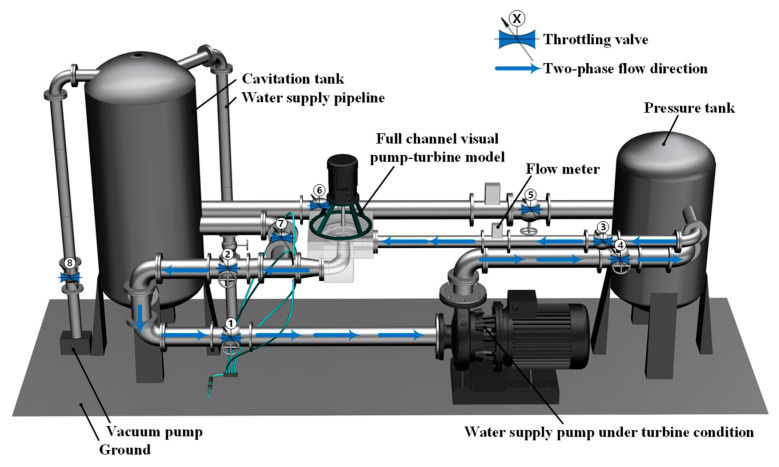
Visualized Pump–Turbine Model Test Bench System (Schematic Diagram).

The experimental setup for investigating pump-turbine performance under pump operating conditions is shown in Figure 4 and Figure 5. The main components include a cavitation tank, vacuum pump, transparent acrylic test section, electromagnetic flowmeter, pressure tank, water supply pump, and pipelines. During the tests, the rotational speed was controlled by a frequency converter, and the inlet flow rate in turbine mode was adjusted by varying the water supply pump speed. Once steady conditions were reached, the inlet and outlet pressure signals at identical turbine points were recorded repeatedly, and the arithmetic average was taken as the result.

**Figure 6:**
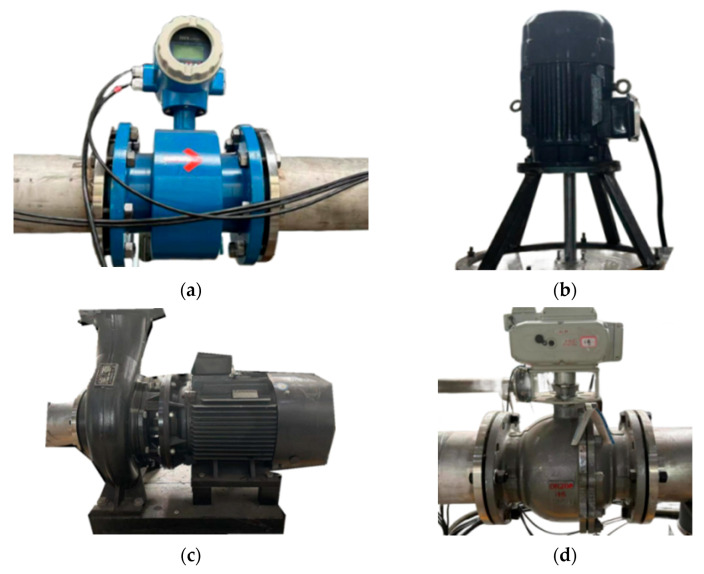
Schematic diagram of some test equipment. (**a**). Electromagnetic Flowmeter (**b**). Three-phase Asynchronous Motor (**c**). Water Supply Pump (**d**). Electromagnetic Ball Valve.

In this process, pressure and flow rate were measured by an electrical parameter meter, pressure sensors, and a high-precision electromagnetic flowmeter. Apparatus specifications are summarized in Table 4, while partial views of the setup appear in Figure 6. After the tests, the results were analyzed using different data-processing approaches to study unsteady flow characteristics of the pump-turbine under turbine conditions. Finally, the experimental data were compared with numerical simulations to confirm the feasibility and accuracy of the method. Multidimensional sensor measurements are listed in Table 5.

To ensure the accuracy of the numerical simulations, this study adopts a combined approach of numerical simulation and experimental measurement. The experimental results are used to validate the calculated unit speed and unit flow rate, thereby further verifying the reliability of the simulation. The parameters of the pump-turbine are described using the dimensionless quantities of unit speed $n_{11}$ and unit flow rate $Q_{11}$. Specifically, the unit speed $n_{11}$ refers to the rotational speed of a geometrically similar pump-turbine with a runner diameter of 1 m and an effective head of 1 m, while the unit flow rate $Q_{11}$ represents the effective flow rate of such a geometrically similar pump-turbine under the same conditions. Consequently, these two unit parameters provide an intuitive means to validate the hydraulic characteristics of the pump-turbine operating in turbine mode.

(2) $$n_{11}=\frac{nD_{1}}{\sqrt{H}}$$

(3) $$Q_{11}=\frac{Q}{D_{1}^{2}\sqrt{H}}$$

**Figure 7:**
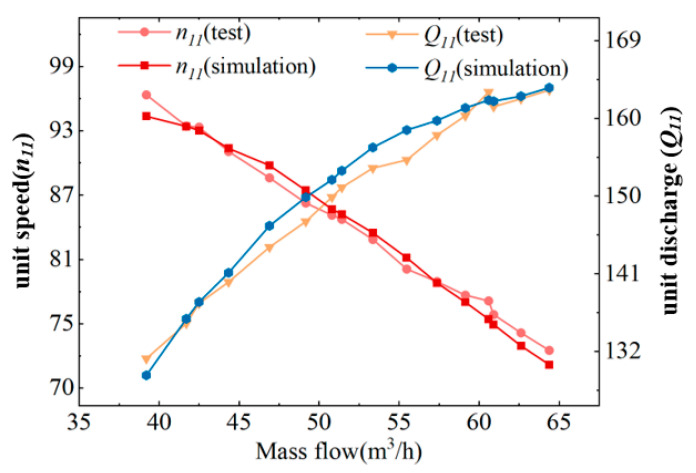
Comparison of Numerical Simulation and Experimental Results for External Turbine Characteristics.

The key parameters of the pump-turbine were measured using the experimental rig and equipment shown in Figure 4, Figure 5 and Figure 6. The calculated and analyzed results of the external characteristic tests and numerical simulations are presented in Figure 7. A good agreement is observed between the simulation and experimental results, with the maximum deviation being less than 3%. Under the design condition, the experimentally measured unit speed and unit flow rate were 74.59 and 162.19, respectively, while the simulated values were 75.10 and 163.30, yielding a final error of 0.69%. Under other operating conditions, the maximum error was 2.81%.

## 3. Mathematical Model

As a type of hydraulic machinery, the internal fluid flow in a pump-turbine is governed by fundamental physical principles, specifically the continuity, momentum, and energy equations [28].

In this study, the Euler–Lagrange approach was employed to perform numerical simulations of the solid–liquid–gas three-phase flow in a pump-turbine. In the numerical model, the Mixture model is based on the assumption of a single velocity and pressure field, making it suitable for multiphase flows that are either fully mixed or partially separated. It solves the mass, momentum, and energy conservation equations in a coupled manner to compute the averaged characteristics of the multiphase flow, while introducing a slip velocity to describe the relative motion between phases. The Mixture model provides a reasonable prediction of velocity and volume fraction distributions in three-phase flows while balancing computational cost and accuracy [29]. The standard SST k–ω (Shear Stress Transport k–ω) model is adopted to improve the prediction of turbulent separation and recirculation zones, enhancing the accuracy of turbulence simulation [30,31,32]. The Generic erosion model is used for quantitative prediction of wear on flow-passage component surfaces [33].

Continuity Equation:

(4) $$\frac{\partial }{\partial_{t}}\left(\rho_{m}\right)+\nabla \cdot \left(\rho_{m}{\vec{V}}_{m}\right)=\overset{•}{m}$$

where $\rho_{m}$ denotes the mass-averaged density of the mixture phase (kg/m^3^); $\overset{\to }{V_{m}}$ denotes the mass-averaged velocity of the mixture phase (m/s).

Momentum Equation:

(5) $$\begin{array}{l} \frac{\partial }{\partial_{t}}\left(\rho_{m}{\vec{V}}_{m}\right)+\nabla \cdot \left(\rho_{m}{\vec{V}}_{m}{\vec{V}}_{m}\right)=-\nabla \rho +\nabla \cdot \left[\mu_{m}\left(\nabla {\vec{V}}_{m}+\nabla {\vec{V}}_{m}^{T}\right)\right]\\ +\rho m\vec{g}+\vec{F}+\nabla \cdot \left(\sum_{k=1}^{n}\alpha_{k}\rho_{k}{\vec{V}}_{dr,k}{\vec{V}}_{dr,k}\right) \end{array}$$

where $\overset{\to }{F}$ denotes the body force (N/m^3^); ${\vec{V}}_{dr,k}$ denotes the drift velocity of phase *k* (m/s).

Energy Equation:

(6) $$\frac{\partial }{\partial_{t}}\sum_{k=1}^{n}\left(\alpha_{k}\rho_{k}E_{k}\right)+\nabla \cdot \sum_{k=1}^{n}\left[\alpha_{k}{\vec{V}}_{k}\left(\rho_{k}E_{k}+p\right)\right]=\nabla \cdot \left(k_{eff}\nabla T\right)+S_{E}$$

where $k_{eff}$ denotes the effective thermal conductivity; $S_{E}$ denotes the volumetric heat source (W/m^3^).

(7) $$\frac{\partial }{\partial_{t}}\left(\alpha_{k}\rho_{k}\right)+\nabla \cdot \left(\alpha_{k}\rho_{k}{\vec{V}}_{m}\right)=-\nabla \cdot \left(\alpha_{k}\rho_{k}{\vec{V}}_{dr,k}\right)$$

Relative (Slip) Velocity and Drift Velocity:

The relative velocity is defined as the velocity of the secondary phase p relative to phase q.

(8) $${\vec{V}}_{qp}={\vec{V}}_{p}+{\vec{V}}_{q}$$

The drift velocity can then be expressed as:

(9) $${\vec{V}}_{dr,p}={\vec{V}}_{qp}-\sum_{k=1}^{n}\frac{\alpha_{k}\rho_{k}}{\rho_{m}}{\vec{V}}_{qk}$$

The SST k−ε turbulence equations are as follows:

(10) $$\frac{\partial (\rho k)}{\partial t}+\frac{\partial (\rho k\bar{u_{i}})}{\partial t}=\frac{\partial \left(\Gamma k\frac{\partial k}{\partial x_{j}}\right)}{\partial x_{j}}+F_{k}-\gamma_{k}$$

(11) $$\frac{\partial (\rho \omega )}{\partial t}+\frac{\partial (\rho \omega \bar{u_{i}})}{\partial x_{i}}=\frac{\partial \left(\Gamma \omega \frac{\partial \omega }{\partial x_{i}}\right)}{\partial x_{i}}+F_{\omega }-\gamma_{\omega }+D_{\omega }$$

where *k* represents the turbulent kinetic energy; *t* denotes time; *ρ* signifies density (kg/m^3^); $\bar{u_{i}}$ indicates the mean turbulent velocity; *x* stands for coordinate components; *ω* denotes the turbulence dissipation rate; $\Gamma_{k}$ and $\Gamma_{\omega }$ represent effective diffusion coefficients; $F_{k}$ and $F_{\omega }$ are the turbulence production terms; $Y_{k}$ and $Y_{\omega }$ signify the dissipation terms for *k* and *ω*; and $D_{\omega }$ represents the diffusion term.

The generic erosion model average erosion rate (*Eg*) and accretion rate ($R_{accretion}$) calculated via the generic erosion model are as follows:

(12) $$E_{g}\;=\;\sum_{p=1}^{N_{\mathrm{particles}}}\frac{{\dot{m}}_{p}C\left(d_{p}\right)f\left(\gamma \right)v^{b\left(v\right)}}{A_{\mathrm{face}}}$$

(13) $$C{(d}_{p})=1.559e^{-6}B^{-0.59}\;\times \;\phi$$

(14) $$v^{b\left(v\right)}=v^{1.73}$$

(15) $$f(\gamma )=\{\begin{array}{ll} 0+22.7\gamma \;-\;38.7\gamma^{2} & \gamma \;\leq \;0.267\;\mathrm{rad}\\ 2+6.8\gamma \;-7.5\gamma^{2}+2.25\gamma^{3} & \gamma \;>\;0.267\;\mathrm{rad} \end{array}\}$$

(16) $$R_{\mathrm{accretion}}=\sum_{p=1}^{N_{\mathrm{particles}}}\frac{{\dot{m}}_{p}}{A_{\mathrm{face}}}$$

where $E_{g}$ represents the erosion rate, kg/m^2^s; $R_{\mathrm{accretion}}$ represents the accretion rate, kg/m^2^s; $d_{p}$ represents a function of the particle diameter; γ denotes the particle impact angle (rad); $f\left(\gamma \right)$ is the relative velocity of particles; v is the impact angle function; $b\left(v\right)$ denotes the relative velocity function of the particles; $A_{\mathrm{face}}$ represents the area of the wall face where the particle strikes the boundary, m^2^; ${\dot{m}}_{p}$ is the mass flow rate of particles colliding with the wall surface, kg/s; ϕ denotes the particle shape factor coefficient (1.0 for angular/angular sand, 0.53 for semicircular sand, and 0.2 for full circular sand, used in this study). In this work, fully circular sand grains are assumed, as they closely resemble the natural quartz sand typically found in southwest China’s rivers, and B represents the Brinell hardness of the material. For acrylic glass, a widely used material in sediment erosion studies due to its transparency and well-documented properties, B = 20.

## 4. Results and Discussion

### 4.1. Air Injection Scheme for Guide Vanes

#### 4.1.1. Velocity Analysis at Different Air Injection Concentrations

**Figure 8:**
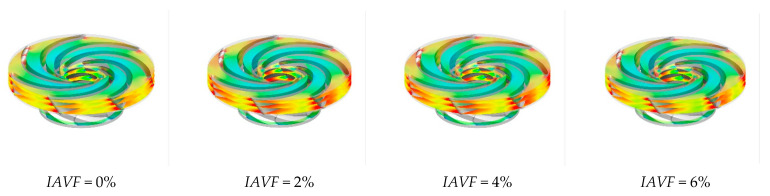
Slicing speed of runner with different aeration concentrations.

**Figure 9:**
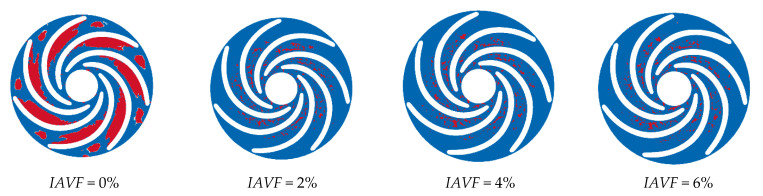
Vorticity contours of runner cross-sections under different air injection concentrations.

**Figure 10:**
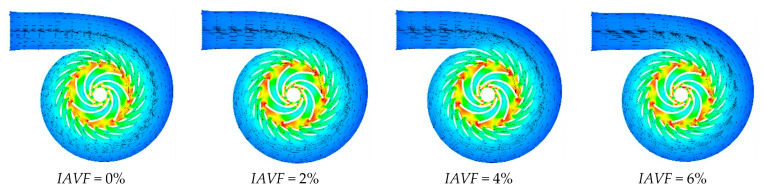
Velocity vector plots of runner cross-sections under different air-entrainment concentrations.

As shown in Figure 8, Figure 9 and Figure 10, when IAVF = 0%, the velocity distribution at the runner inlet is relatively uniform and stable. As IAVF increases to 2%, the inlet velocity rises significantly, due to the bubbles moving faster than the water, which induces a traction effect on the flow field. With further increases to 4% and 6%, the inlet velocity gradually decreases, resulting from the combined effects of increased bubble content, higher flow resistance, and enhanced turbulent dissipation. Under high IAVF conditions, vortex structures are intensified, leading to greater energy loss and reduced main flow velocity. Overall, low-concentration aeration helps to enhance flow velocity, whereas high-concentration aeration reduces velocity due to increased energy dissipation. Meanwhile, the outlet velocity of the runner gradually increases with rising IAVF.

**Figure 11:**
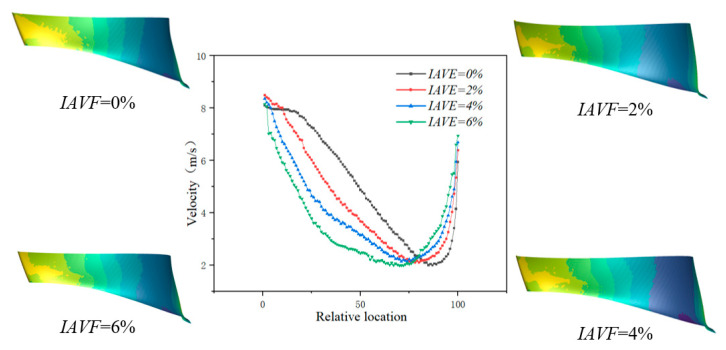
The velocity curve of the pressure surface was extracted along the chord length of the blade with IAVF.

Figure 11 shows the velocity variation along the pressure surface of the blade at different air injection concentrations. As illustrated, when IAVF = 0%, the velocity near the leading edge of the blade is relatively high. After air injection IAVF > 0%, the leading-edge velocity further increases, but the rate of increase gradually diminishes with higher aeration levels. In contrast, the mid-span velocity of the blade significantly decreases after air injection, with the reduction becoming more pronounced as IAVF increases, reaching the maximum decrease at IAVF = 6%. At IAVF = 0%, the trailing-edge velocity is the lowest; with increasing IAVF, the trailing-edge velocity gradually rises and attains its maximum at IAVF = 6%.

**Figure 12:**
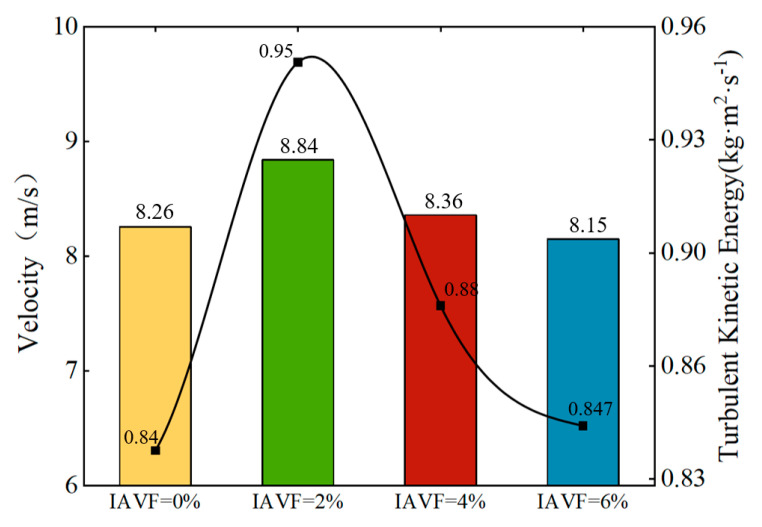
The maximum pressure surface velocity and turbulent kinetic energy of different IAVF blades.

As shown in Figure 12, both the velocity and turbulent kinetic energy on the blade pressure side exhibit a “rise then fall” trend with increasing inlet air volume fraction (IAVF). At IAVF = 2%, the pressure-side velocity peaks at 8.84 m/s, and the turbulent kinetic energy reaches 0.95 kg·m^2^/s^2^. With further increases in IAVF, both gradually decrease, reaching 8.15 m/s and 0.847 kg·m^2^/s^2^ at IAVF = 6%.

This trend can be attributed to the mechanisms of bubbles at different concentrations. At low concentrations (0~2%), the bubbles reduce the local fluid density upon entering the flow passage, improving fluid mobility and facilitating the conversion of energy into kinetic energy. Simultaneously, bubble-induced disturbances enhance flow instability and promote turbulence development, resulting in a significant increase in turbulent kinetic energy. At higher concentrations (≥4%), the number of bubbles increases markedly, strengthening their relative velocity and interactions with the liquid phase. This leads to elevated local fluid viscosity and more complex turbulent structures. Some bubbles may coalesce or collapse, increasing dissipation, while the “blocking effect” of bubbles in the passage further augments flow resistance. As a result of these combined effects, both the maximum velocity and turbulent kinetic energy on the blade pressure side decrease significantly.

**Figure 13:**
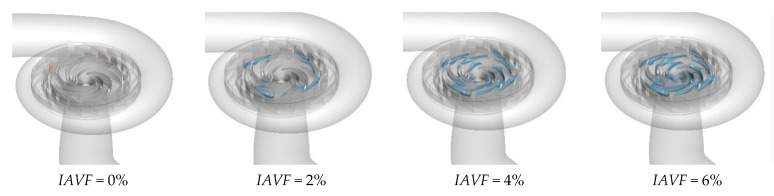
The equivalent surface of the gas volume fraction of 0.1 with different aeration concentrations at 1.2 s.

As shown in Figure 13, the distribution of the iso-surface with a gas volume fraction of 0.1 in the runner passage exhibits significant changes with increasing IAVF. When IAVF = 2%, only a small number of bubbles enter the passage, mainly concentrated in localized regions and distributed along the streamlines under fluid-induced motion, without fully occupying the passage. As IAVF increases to 4%, more bubbles enter the passage, and their distribution within the runner expands. Enhanced vortex and shear effects lead to a more uniform bubble distribution and a wider coverage area. When IAVF further increases to 6%, the higher aeration concentration results in a dense accumulation of bubbles, which tend to occupy the entire runner passage.

#### 4.1.2. Analysis of Runner Velocity Under Different Air Injection Concentrations

**Figure 14:**
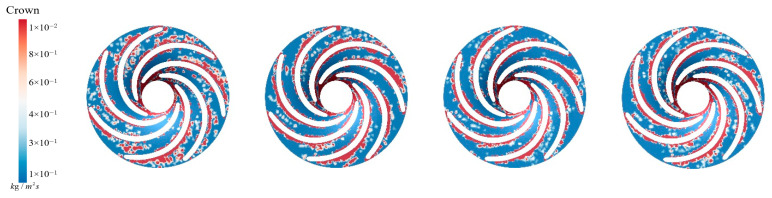
Crown sediment deposition under different aeration concentrations.

As shown in Figure 14, under no aeration IAVF = 0%, sediment deposition is relatively dispersed and randomly distributed throughout the passage. When IAVF = 2%, sediment begins to accumulate on the blade working surfaces, although the overall deposition remains high, indicating that the initial aeration has limited effect on sediment deposition. At IAVF = 4%, sediment accumulation becomes more pronounced, while the total deposition decreases further, suggesting that aeration increasingly influences sediment transport and deposition. At IAVF = 6%, the deposition area is significantly reduced, and the overall sediment amount is minimized, indicating that high aeration concentration effectively suppresses sediment accumulation on the crown. As shown clearly in Figure 14, after aeration, for the upper crown, the buoyancy of bubbles is enhanced, providing stronger lift to sediment particles, with some particles being re-entrained into the main flow, thereby reducing deposition. The introduction of bubbles also increases the turbulence intensity in the flow, causing stronger perturbations to particle trajectories, which makes it difficult for large deposition areas to form within the passage.

**Figure 15:**
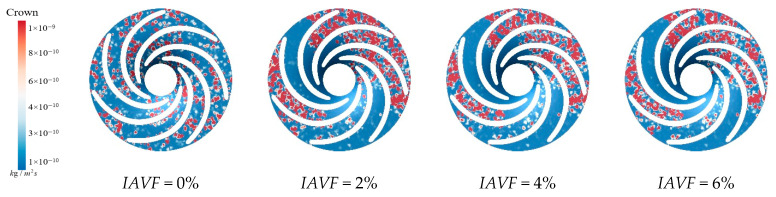
Sediment erosion on the crown of the runner under different aeration concentrations.

Figure 15 illustrates the sediment erosion on the runner crown under different aeration concentrations. Without aeration, IAVF = 0%, sediment erosion is relatively dispersed across the crown, with some flow passages experiencing more severe wear. As the aeration concentration increases, IAVF = 2%, 4%, 6%, the overall erosion on the crown gradually decreases, with erosion primarily concentrated in certain flow passages while other regions experience significantly less wear. Simultaneously, the sediment deposition on the crown surface also decreases with increasing aeration, especially at IAVF = 4% and 6%, where the total sediment deposition is significantly reduced. As shown in Figure 15, aeration reduces sediment deposition on the upper cover surface, fundamentally decreasing the degree of sediment-induced wear. As the aeration concentration increases, the presence of bubbles carries a portion of sediment particles further downstream, preventing them from depositing on the upper cover surface. This results in a reduction in sediment accumulation on the upper cover, thereby mitigating erosion and wear.

**Figure 16:**
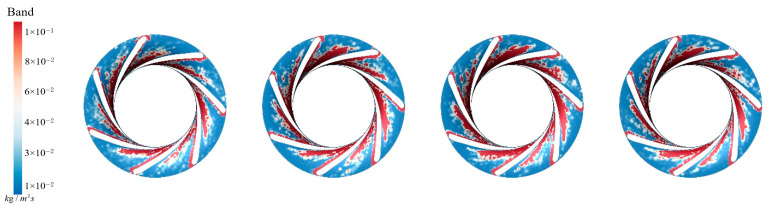
Sediment deposition on the lower ring of the runner under different aeration concentrations.

As shown in Figure 16, under no aeration, IAVF = 0%, sediment deposition is randomly distributed and mainly concentrated near the blade regions. When IAVF = 2%, the deposition area expands compared to the no-aeration case, although the overall distribution remains relatively uniform. At IAVF = 4%, sediment becomes more concentrated, particularly near the blade working surfaces. With IAVF = 6%, sediment deposition is most pronounced, indicating that high aeration concentrations promote sediment accumulation within the lower band. Figure 16 illustrates that aeration affects the vortex characteristics within the blade passage, enlarging the low-velocity recirculation zones, which makes sediment more likely to accumulate and deposit in these areas. Additionally, aeration increases the pressure gradient near the guide vane working surface, causing the resultant force on sediment particles in the flow field to direct toward this region. Consequently, sediment gradually concentrates near the guide vane working surface.

**Figure 17:**
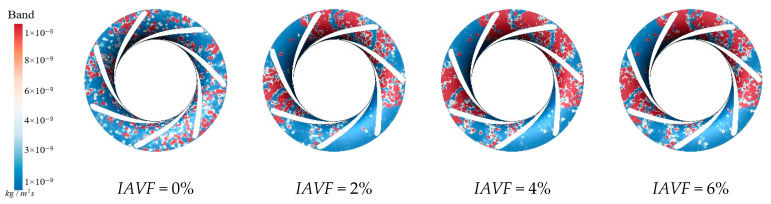
Sediment erosion on the lower ring of the runner under different aeration concentrations.

Figure 17 presents the sediment erosion distribution on the runner lower band under different aeration concentrations, IAVF. Without aeration, IAVF = 0%, sediment erosion is relatively uniformly distributed on the lower band surface, exhibiting a random pattern. As the aeration concentration increases, IAVF = 2%, 4%, 6%, erosion gradually concentrates in specific flow passages, and the erosion intensity increases. The trend shown in Figure 17 is consistent with the previously analyzed changes in sediment deposition in the lower shroud; that is, as the IAVF increases, the sediment deposition in the lower shroud also increases, leading to an intensification of local wear.

**Figure 18:**
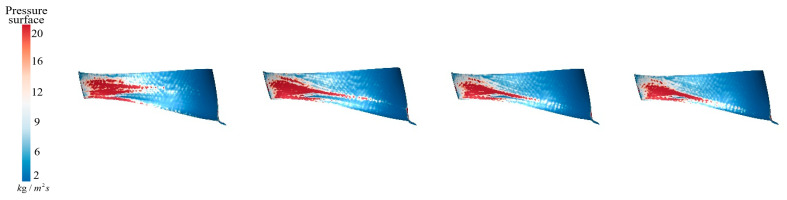
Sediment deposition on the pressure surface of the runner under different aeration concentrations.

Figure 18 shows the sediment deposition on the pressure surface of the runner under different aeration concentrations. It can be observed that, without aeration, IAVF = 0%, sediment on the guide vane working surfaces mainly deposits from the blade leading edge to the mid-span region. As the aeration concentration increases, IAVF = 2%, 4%, 6%, sediment tends to accumulate toward the lower part of the blade. However, although sediment gradually shifts toward the blade lower region, the deposition at the blade leading edge and mid-span region does not decrease significantly, indicating that aeration alters the sediment distribution without effectively reducing deposition in the original regions.

**Figure 19:**
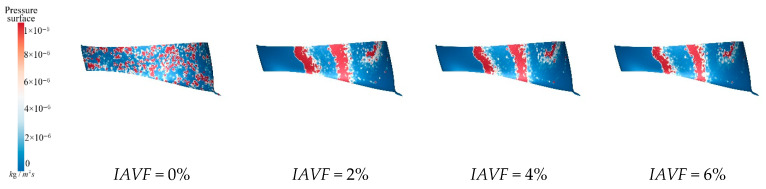
Sediment erosion on the pressure surface of the runner under different aeration concentrations.

As shown in Figure 19, under no aeration, IAVF = 0%, sediment erosion is also relatively dispersed across the entire working surface. With increasing aeration concentration, IAVF = 2%, 4%, 6%, erosion gradually concentrates in the mid-to-rear regions of the blade, while erosion at the leading part is significantly reduced. At the same time, the overall erosion intensity gradually weakens, decreasing with increasing aeration concentration. This phenomenon is consistent with the observed decrease in blade surface velocity, indicating that for runner blades, the impact of aeration on the flow field characteristics is the main factor influencing erosion variations.

**Figure 20:**
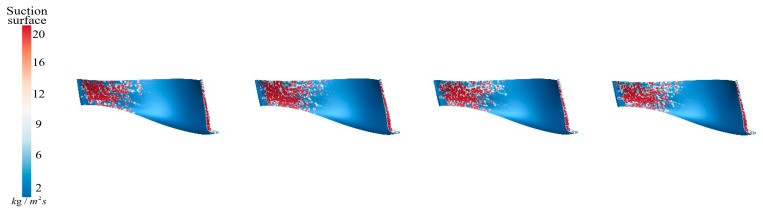
Sediment erosion on the pressure surface of the runner under different aeration concentrations.

As shown in Figure 20, the introduction of bubbles alters the local pressure gradients and turbulence characteristics, particularly in the lower regions of the blade, which may induce localized recirculation or low-velocity zones, making sediment more prone to retention and deposition. In addition, aeration affects the trajectories of sediment particles. At higher aeration concentrations, bubbles promote sediment accumulation toward the lower blade regions. Meanwhile, the flow characteristics at the blade leading edge and mid-span remain fundamentally unchanged, retaining a high capacity for sediment capture, so the deposition in these regions does not decrease significantly.

**Figure 21:**
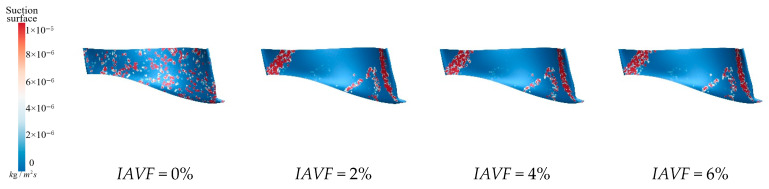
Sediment erosion on the suction surface of the runner under different aeration concentrations.

Figure 21 illustrates the sediment erosion distribution on the suction surface of the runner under different aeration concentrations, IAVF. Without aeration (IAVF = 0%, sediment erosion is relatively dispersed across the suction surface, with randomly distributed erosion locations. As the aeration concentration increases, IAVF = 2%, 4%, 6%, erosion gradually concentrates in the lower parts of the leading and trailing edges, while erosion in other regions is significantly reduced. This redistribution indicates that aeration causes sediment to accumulate near the leading and trailing edges, shifting the impact locations on the suction surface and forming localized high-erosion areas.

### 4.2. Shaft Air Injection Scheme

#### 4.2.1. Effect of Different Air Injection Concentrations on Runner Velocity

**Figure 22:**
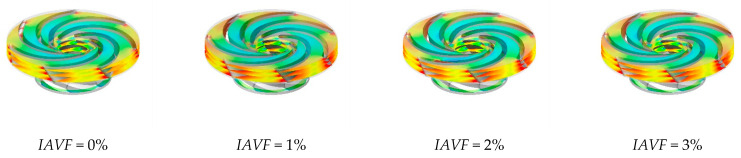
Slicing speed of runner with different aeration concentrations.

**Figure 23:**
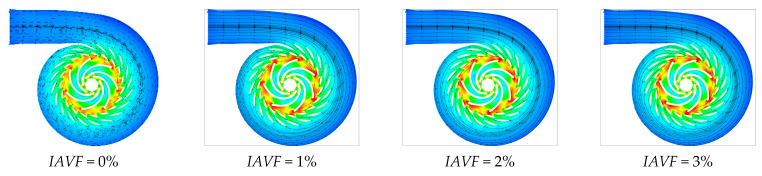
Vorticity contours of runner cross-sections under different air injection concentrations.

As shown in Figure 22 and Figure 23, when IAVF = 0%, the velocity in the blade-free region adjacent to the adjustable guide vanes up to the runner inlet is relatively uniform. With increasing aeration, IAVF = 1%, 2%, 3%, the velocity in this region significantly increases, and localized high-velocity zones may appear. In contrast to the velocity rise at the inlet, the low-velocity regions within the runner passage gradually expand toward the blade working surfaces with increasing aeration, particularly on the suction side, manifesting as a reduction in high-velocity areas and an overall shift in the flow field toward lower velocities.

At the runner inlet, bubbles carry higher momentum, which may reduce local pressure and accelerate fluid entry into the runner. However, within the flow passage, the bubbles decrease the effective density and alter the pressure gradient, lowering the efficiency of kinetic energy conversion and expanding low-velocity regions. Although the high-speed inflow absorbs more kinetic energy at the inlet, aeration also induces energy dissipation, preventing part of the energy from being transferred to the blade passages and further enlarging the low-velocity zones. The expansion of these zones may reduce the impact velocity of sediment particles on the working surface, thereby mitigating erosion and wear.

**Figure 24:**
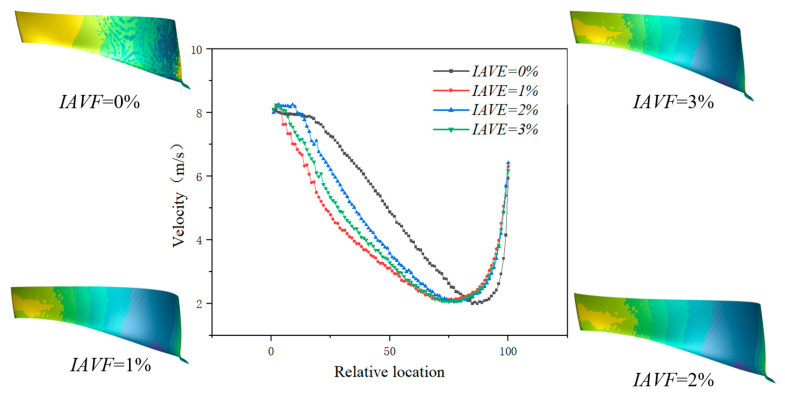
The velocity curve of the pressure surface was extracted along the chord length of the blade with IAVF.

As shown in Figure 24, the velocity profiles along the blade pressure side under different IAVF conditions exhibit distinct variations. At the leading-edge region, the velocity with aeration is consistently higher than that without aeration (IAVF = 0%), indicating a pronounced local acceleration effect at the blade inlet. However, as the flow develops into the 10–20% chord section near the leading edge, the velocity with aeration decreases rapidly and falls below that of the non-aerated case. In the mid-blade region, the velocity gradually decreases with increasing aeration concentration, with the reduction being more pronounced at higher IAVF values (2% and 3%). This is primarily attributed to the relative slip effect between bubbles and liquid, which increases flow resistance and energy dissipation. Approaching the trailing-edge region, the velocity recovers and becomes slightly higher than in the non-aerated condition.

**Figure 25:**
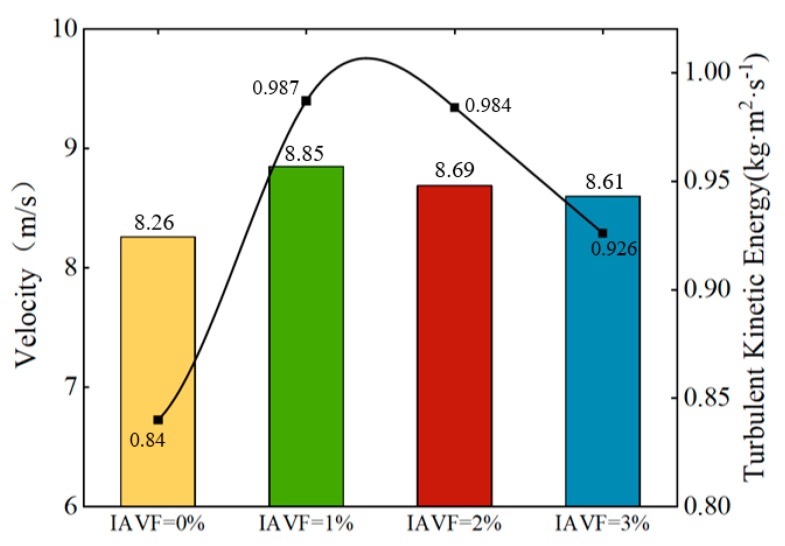
The maximum pressure surface velocity and turbulent kinetic energy of different IAVF blades.

As shown in Figure 25, the maximum velocity and turbulent kinetic energy on the blade pressure surface under different IAVF conditions exhibit distinct trends. With the introduction and gradual increase in aeration, the maximum velocity on the blade pressure surface initially increases and then gradually decreases. Specifically, at IAVF = 1%, the maximum velocity reaches 8.85 m/s, showing a significant increase compared to the no-aeration case, IAVF = 0%, 8.26 m/s. As IAVF further increases to 2% and 3%, the maximum velocity decreases to 8.69 m/s and 8.61 m/s, respectively, indicating that higher aeration concentrations gradually reduce the peak velocity on the pressure surface.

Similarly, the turbulent kinetic energy exhibits a comparable trend. Without aeration, the turbulent kinetic energy is relatively low (0.84 kg·m^2^·s^−2^). When aeration is introduced at IAVF = 1%, the turbulent kinetic energy sharply rises to its maximum value of 0.987 kg·m^2^·s^−2^. With further increases in aeration to 2% and 3%, the turbulent kinetic energy slightly decreases to 0.984 kg·m^2^·s^−2^ and 0.926 kg·m^2^·s^−2^, respectively.

#### 4.2.2. Effect of Different Air Injection Concentrations on Runner Sediment Erosion

**Figure 26:**
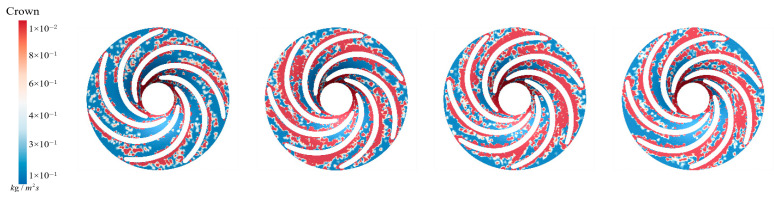
Crown sediment deposition under different aeration concentrations.

As shown in Figure 26, both the distribution and intensity of sediment deposition on the runner crown change significantly under different IAVF conditions. Without aeration (IAVF = 0%), the deposition is relatively uniform and dispersed throughout the flow passage. With increasing IAVF, however, the sediment gradually concentrates near the guide vane working surface and forms localized accumulation zones. The total amount of deposition exhibits a trend of first increasing and then decreasing as IAVF rises from 0% to 1%, 2%, and 3%, with the most severe accumulation occurring at 1% and 2%, and slightly alleviated at 3%. In the initial non-aerated condition, the flow remains relatively stable, and particles are evenly distributed under fluid forces. With aeration, the interactions between bubbles and liquid intensify, leading to variations in local turbulent kinetic energy and velocity gradients. In particular, larger low-velocity regions emerge in the mid-passage, which enhances particle collisions and agglomeration, making them more likely to concentrate and deposit near the guide vane working surface.

**Figure 27:**
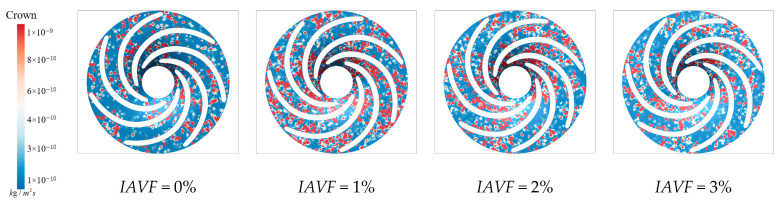
Sediment erosion on the crown of the runner under different aeration concentrations.

As shown in Figure 27, at IAVF = 0%, sediment-induced wear regions are relatively dispersed, and the crown experiences varying degrees of erosion. With aeration at IAVF = 1%, the spatial distribution changes little, but wear intensity rises sharply, especially near the blade inlet, where high-wear zones expand. At IAVF = 2%, wear intensity decreases and the high-wear region shrinks, indicating reduced sediment impact. At IAVF = 3%, wear is further alleviated, closely associated with deposition changes: initially dispersed deposition converges into larger accumulations, while higher aeration partly mitigates it. These results indicate a close correlation: significant increases in sediment deposition correspond to intensified erosion distribution, while reductions in deposition are accompanied by decreased erosion on the upper band.

**Figure 28:**
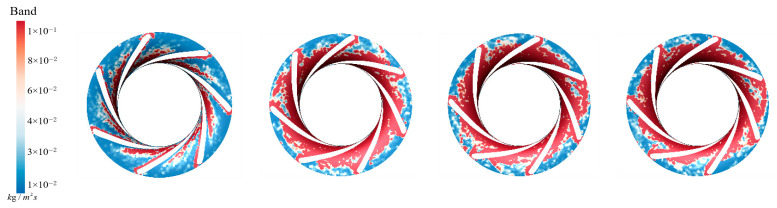
Sediment deposition on the lower ring of the runner under different aeration concentrations.

As shown in Figure 28, the sediment deposition on the runner lower band under different IAVF conditions exhibits a pronounced increase with the introduction and gradual rise in aeration. In the no-aeration state, IAVF = 0%, sediment deposition within the passage is relatively sparse and dispersed. When the aeration concentration increases to 1%, sediment deposition becomes significantly enhanced, forming more distinct and dense accumulation regions. With further increases to 2% and 3%, sediment deposition intensifies, as indicated by the expansion of covered areas and a marked increase in deposition density, particularly near the blade working surfaces. In the lower band region of the runner blades, the effect of gravity facilitates the settling and accumulation of sediment particles in this area.

**Figure 29:**
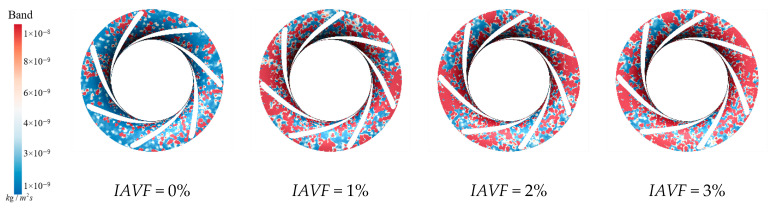
Sediment erosion on the lower ring of the runner under different aeration concentrations.

As shown in Figure 29, without aeration, IAVF = 0%, sediment erosion on the lower band is relatively dispersed, with overall low intensity and only mild erosion in localized regions. With the introduction of aeration, IAVF = 1%, erosion intensity significantly increases, particularly near the passage inlet, where the high-erosion zones expand markedly. This indicates that higher aeration concentrations impart greater kinetic energy to sediment particles, intensifying their impact and erosion on the lower band surface. As the aeration concentration further increases to 2% and 3%, erosion intensity continues to rise, with localized regions experiencing pronounced expansion, reflecting more severe wear. At lower aeration concentrations, sediment particles are dispersed and possess relatively low kinetic energy, resulting in scattered deposition and mild erosion. With higher aeration concentrations, particles gain stronger impact forces, leading to more concentrated deposition on the blade surfaces and consequently exacerbating erosion.

**Figure 30:**
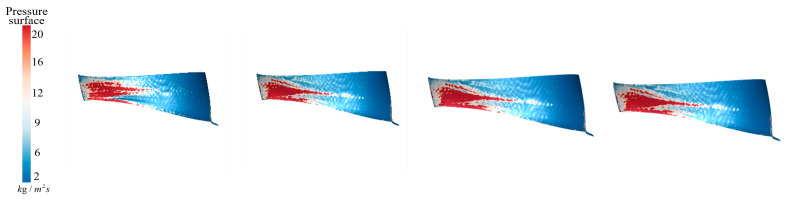
Sediment deposition on the pressure surface of the runner under different aeration concentrations.

As shown in Figure 30, with IAVF gradually increasing from 0% to 1%, 2%, and 3%, the primary sediment deposition on the runner blade working surfaces shifts slightly from the initial concentration near the blade leading edge and mid-section toward the lower part of the blade. However, it is noteworthy that the original deposition at the leading edge and mid-section does not significantly decrease across these aeration conditions. Therefore, simply increasing IAVF does not effectively reduce sediment accumulation in the leading and mid-regions. With higher aeration, new low-velocity or recirculation zones may form near the lower portion of the guide vanes due to altered pressure gradients. Under the coupled effects of the gas–liquid phase and sediment particles, sediment gradually migrates downward and accumulates again, resulting in pronounced deposition in the lower regions.

**Figure 31:**
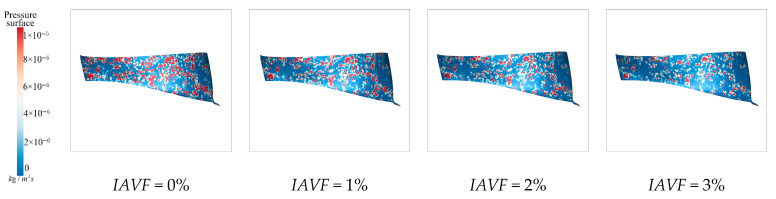
Sediment erosion on the pressure surface of the runner under different aeration concentrations.

As shown in Figure 31, without aeration, LAVF = 0%, sediment erosion on the blade working surfaces is relatively dispersed, with only mild localized wear and no distinct concentrated regions. With the introduction of aeration, LAVF = 1%, the distribution and intensity of erosion remain largely unchanged. For the blade working surfaces, the addition of aeration does not significantly alter sediment deposition and therefore, does not affect the intensity of erosion distribution. When LAVF further increases to 2% and 3%, erosion is slightly alleviated.

As discussed in the previous section, increasing aeration concentration leads to a reduction in flow velocity on the blade working surfaces. The lower velocity decreases the impact force of sediment particles on the blade, thereby slowing the erosion process. Since sediment deposition has minimal influence on erosion under these conditions, this effect is primarily attributed to the reduced flow velocity: lower velocity decreases the kinetic energy of the sediment particles, mitigating their impact on the blade surfaces and consequently alleviating erosion. Therefore, the observed changes in blade surface erosion are closely related to flow velocity variations, while changes in sediment deposition do not significantly affect erosion intensity.

**Figure 32:**
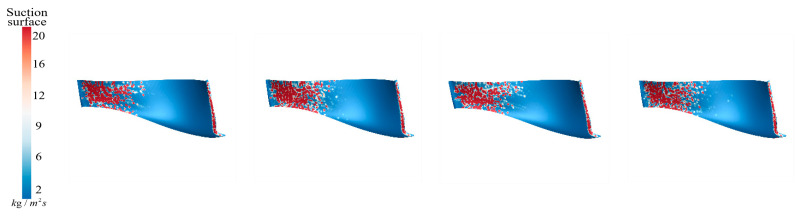
Sediment erosion on the pressure surface of the runner under different aeration concentrations.

As shown in Figure 32, even with LAVF gradually increasing from 0% to 3%, the sediment deposition pattern on the suction surfaces of the runner blades does not exhibit significant changes. Sediment remains primarily concentrated near the blade leading edge and parts of the blade body, and no notable reduction or large-scale migration is observed with increasing aeration concentration.

**Figure 33:**
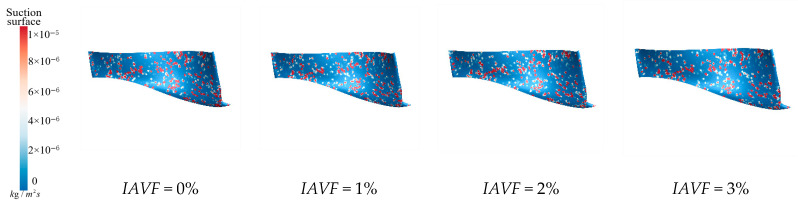
Sediment erosion on the suction surface of the runner under different aeration concentrations.

As shown in Figure 33, the sediment erosion distribution on the suction surfaces of the runner blades under different LAVF conditions exhibits minimal changes in spatial distribution as the aeration concentration increases. This trend is consistent with the sediment deposition patterns, where the deposition regions and distributions do not undergo significant alterations. In particular, under LAVF = 1%, 2%, and 3%, the increase in sediment deposition on the suction surfaces is not significant, and the expansion of erosion regions is relatively limited.

**Figure 34:**
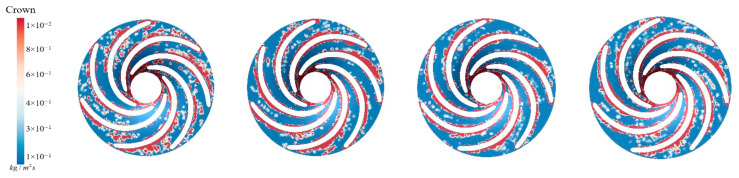
Sediment deposition distribution on the crown of the runner under different aeration concentrations.

**Figure 35:**
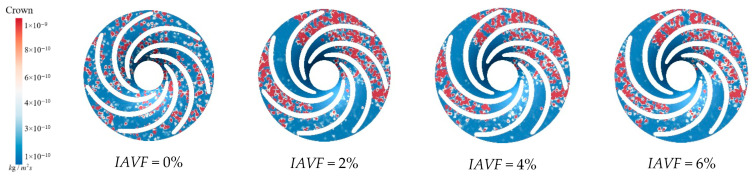
Sediment erosion distribution on the crown of the runner under different aeration concentrations.

**Figure 36:**
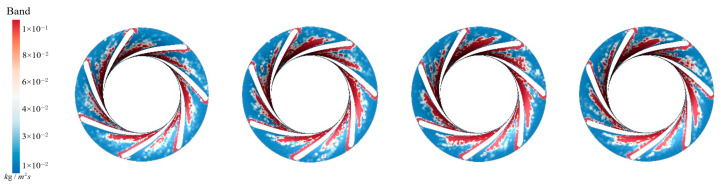
Sediment deposition distribution on the lower ring of the runner under different aeration concentrations.

**Figure 37:**
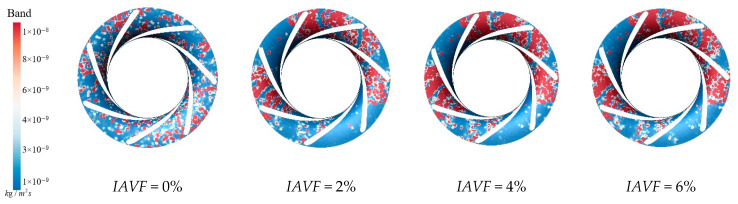
Sediment erosion distribution on the lower ring of the runner under different aeration concentrations.

As shown in Figure 34, Figure 35, Figure 36 and Figure 37, under the operating conditions of guide vane opening 9.3 mm, flow rate 79.2 m^3^/h, rotational speed 1000 r/min, sediment particle diameter 0.001 mm, and concentration 0.1%, the effects of implementing three-phase aeration on the movable guide vanes and main shaft were analyzed. The results indicate that, for the runner crown and lower band regions, aeration can reduce sediment deposition to varying degrees and moderately mitigate erosion within certain areas. This suggests that the aeration bubbles disturb the trajectories of sediment particles in the flow field, weakening direct collisions between particles and wall surfaces, and thereby providing an overall anti-erosion effect.

However, aeration does not alter the characteristic distribution patterns of erosion in the crown and lower band regions. In some severely eroded areas, aeration even leads to slightly intensified erosion and more symmetric distribution patterns. This indicates that, although aeration offers a protective effect, it may locally reinforce the symmetry of the flow field structure, resulting in a more pronounced concentration of high-erosion zones.

## 5. Conclusions

This study systematically investigates the influence of air injection in pump-turbines, with a particular focus on runner wear characteristics—a topic rarely addressed in domestic research. Under unsteady solid–liquid–gas three-phase flow conditions, numerical simulations of particle motion and wear mechanisms were conducted by integrating the Discrete Phase Model (DPM), the SST k–ω turbulence model, the mixture multiphase model, and the Lagrangian approach. The results demonstrate that gas–liquid mixing modifies near-wall flow characteristics, thereby elucidating the governing mechanisms of aeration on wear behavior and its potential for erosion mitigation.

The main conclusions of this study are as follows:

(1) Aeration concentration has a dual effect on flow characteristics. Moderate aeration (IAVF ≤ 2%) promotes energy conversion and enhances flow stability. However, excessive aeration (IAVF ≥ 4%) leads to an increased number of bubbles, resulting in higher resistance, intensified turbulence dissipation, decreased flow velocity, and a “rise–then–fall” trend in turbulent kinetic energy. This indicates that aeration has a positive effect on flow improvement within a certain range, but excessive concentration reduces flow efficiency.
(2) Under guide vane aeration, sediment deposition and wear are significantly influenced by aeration concentration. At low concentration (IAVF = 2%), sediment shifts from the runner crown toward the guide vane surface, and the overall deposition is substantially reduced. When the aeration concentration increases to 6%, sediment on the crown and lower ring decreases markedly, demonstrating the beneficial effect of aeration on sediment transport and deposition. In contrast, shaft aeration increases wear intensity on the crown and lower ring with rising concentration. Although low-concentration aeration can partially alleviate crown wear, at high concentration (IAVF = 3%), the wear region still shows a significant expansion. Overall, guide vane aeration outperforms shaft aeration in erosion mitigation.
(3) Flow velocity has a significant effect on runner blade wear. Changes in the velocity distribution at the blade leading edge and working surface are key factors affecting wear. Low-concentration aeration reduces local wear by decreasing sediment deposition, whereas aeration that increases deposition under certain conditions intensifies wear in corresponding regions. This indicates that the coupling mechanism between velocity fields and deposition patterns is central to explaining wear behavior.
(4) Aeration location effectively mitigates sediment deposition and wear. For the crown and lower ring, aeration enhances the symmetry of the flow field locally, resulting in more concentrated high-wear regions. This study provides theoretical guidance and engineering reference for applying aeration in pump-turbines of pumped-storage power stations, highlighting the importance of optimizing aeration location and concentration.

## Figures and Tables

**Table 1:** Aeration schemes at guide vane (Plan 1).

	IAVF (%)	0	2	4	6
Plan 1	q (m^3^/s)	0	3.4 × 10^−4^	5.1 × 10^−4^	6.8 × 10^−4^
	$q_{m}$ (kg/s)	0	4.165 × 10^−4^	6.2475 × 10^−4^	8.33 × 10^−4^

**Table 2:** Aeration schemes at main shaft (Plan 2).

	IAVF (%)	0	1	2	3
Plan 2	q (m^3^/s)	0	1.7 × 10^−4^	3.4 × 10^−4^	5.1 × 10^−4^
	$q_{m}$ (kg/s)	0	2.0825 × 10^−4^	4.165 × 10^−4^	6.2475 × 10^−4^

**Table 3:** Main geometric parameters of pump turbine model.

Parameters	Unit	Value
Guide vane opening	mm	9.3
Runner blade thickness	mm	8
Runner inlet diameter	mm	200
Runner outlet diameter	mm	115
Draft tube inlet diameter	mm	115
Draft tube outlet diameter	mm	161

**Table 4:** Main parameters of the test rig equipment.

Component	Component Descriptions
Three-phase asynchronous motor	YE2-225S-4; motor power: 37 kW; rated speed: 1480 r/min; rated current: 70.5 A
Horizontal single-stage centrifugal pump	KQW; 200/300S-37/4; motor power: 37 kW; rated speed: 1480 r/min; flow rate: 360 m^3^/h; head: 28 m
Electromagnetic ball valve	Four types used; all motors rated at 150 W; rated currents: 10 A/2.0 A/1.0 A/0.52 A
Electromagnetic flowmeter	Measuring range: 50–250 (unit assumed m^3^/h); accuracy: ±0.5%
Static pressure sensor	BD-801XB; measuring range: −100 to 500 kPa; accuracy: 0.4

**Table 5:** Multidimensional Sensor Test Bench Data Acquisition.

Flow Rate (m^3^/h)	Inlet Pressure RMS (Pa)	Outlet Pressure RMS (Pa)	Inlet Velocity (m/s)	Outlet Velocity (m/s)	Kinetic Energy Difference (m)	Head (m)
97	40,513.56	129,581.5	1.32	1.88	0.09	9.60
95	39,414.01	147,613.9	0.00	0.00	0.00	11.45
92	41,326.07	162,407.3	0.00	0.00	0.00	12.77
90	40,451.41	170,826.6	1.23	1.75	0.08	13.79
87	41,224.26	176,800.9	0.00	0.00	0.00	14.25
85	41,912.71	183,409.9	1.16	1.65	0.07	14.92
80	42,514.48	189,241.1	0.00	0.00	0.00	15.38
75	41,934.06	193,838.1	1.02	1.46	0.05	15.96
72	41,751.5	196,224.4	0.00	0.00	0.00	16.17
70	43,685.06	199,005.4	0.96	1.36	0.05	16.31
65	44,356.18	197,539.1	0.89	1.26	0.04	16.08
60	42,811.17	199,169.4	0.82	1.16	0.03	16.40
55	44,557.35	201,481.6	0.00	0.00	0.00	16.42
50	44,449.51	202,150.4	0.68	0.97	0.02	16.52
45	44,248.92	203,793.9	0.00	0.00	0.00	16.69
40	44,937.42	204,899.7	0.55	0.78	0.02	16.75

## Data Availability

The data that support the findings of this study are available from the corresponding author upon reasonable request.
